# Multi-phase retreat of the Laurentide Ice Sheet and associated freshwater release from Hudson Bay during the last deglaciation

**DOI:** 10.1038/s41598-026-39365-y

**Published:** 2026-03-25

**Authors:** Quentin Duboc, Etienne Brouard, Guillaume St-Onge, Patrick Lajeunesse, Matthias Moros, Kerstin Perner, Martin Roy

**Affiliations:** 1 Institut des sciences de la mer de Rimouski (ISMER), Research Chair in Marine Geology, Université du Québec à Rimouski and GEOTOP, Rimouski (Qc), Canada; 2 Geological Survey of Canada, Natural Resources Canada, Quebec City (QC), Canada; 3 Département des sciences de la Terre et de l’atmosphère, UQAM, Montréal (QC), Canada; 4 Département de géographie, Université Laval, Québec (QC), Canada; 5 Leibniz Institute for Baltic Sea Research Warnemünde, Seestraße 15, Rostock, Germany

**Keywords:** Climate sciences, Hydrology, Solid Earth sciences

## Abstract

During the final retreat of the Laurentide Ice Sheet, large volumes of meltwater from the Hudson Bay region entered the North Atlantic leading to one of the strongest freshwater perturbations of the Holocene. The resulting 8.2 ka cold event is a key benchmark for understanding ice-sheet–ocean interactions, yet the timing, structure, and mechanisms of this freshwater release from Hudson Bay remain debated. New sediment records from Hudson Strait document a sequence of ice–ocean reorganisations and associated freshwater regimes between ~ 9.0 and 8.0 ka. They include (1) background deglacial meltwater and limited iceberg export prior to ~ 8.8 ka; (2) a short-lived episode of marine intrusion, ice-shelf formation, and subsequent ice-shelf breakup accompanied by transient iceberg discharge; and (3) a prolonged, multi-phase freshwater release between ~ 8.2 and 8.0 ka linked to the final collapse of the Hudson Bay ice saddle and drainage of glacial Lake Agassiz–Ojibway. Notably, the duration of this final freshwater phase is coeval, within uncertainty, with the 8.2 ka climate anomaly recorded in Greenland ice cores, consistent with a causal connection. This refined stratigraphic framework demonstrates that freshwater forcing from Hudson Bay was temporally structured and temporally sustained, highlighting the importance of ice-margin geometry and meltwater routing in modulating early Holocene climate perturbations.

**Supplementary Information:**

The online version contains supplementary material available at 10.1038/s41598-026-39365-y.

## Introduction

**Fig. 1:**
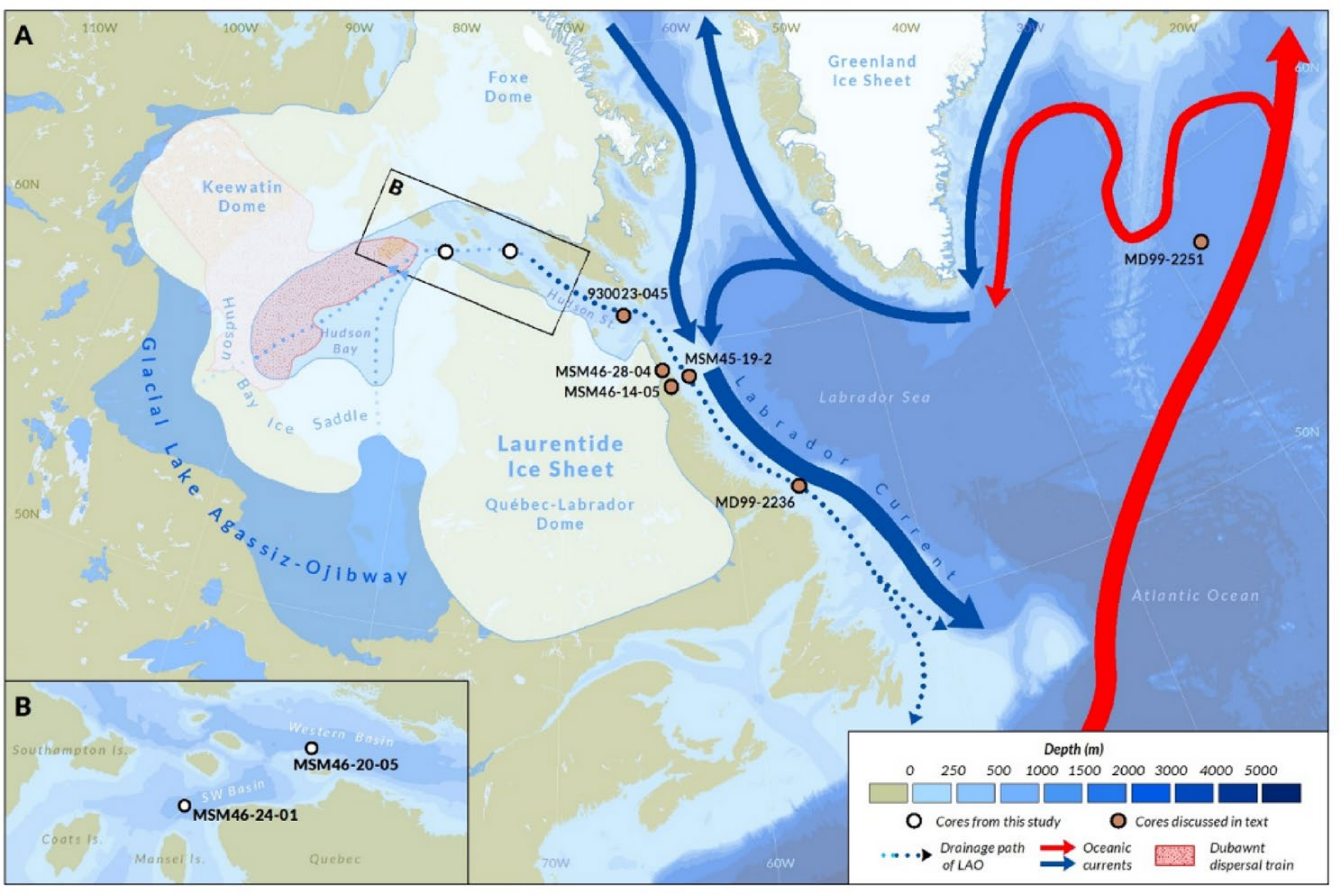
(**A**) Location of the Hudson Strait cores relative to the extent of the Laurentide Ice Sheet, its major domes and saddles, and LAO at ~ 8.7 ka BP^29^. Also shown are drainage pathways of the lake, major ocean circulation patterns, key marine records bearing red beds and radiocarbon chronologies, and the dispersal extent of Dubawnt Formation red till^30,31^. Bathymetry from GEBCO^32^. (**B**). Specific core sites (MSM46-20-05GC and MSM46-24-01GC) and bathymetry of the basins from which they were recovered.

Large variations in ice-sheet extent and associated changes in meltwater discharge profoundly modulated regional climate and ocean circulation during the last deglaciation. Understanding these ice–ocean interactions is therefore essential for assessing the impacts of ongoing and future cryospheric change^1,2^. The most prominent episode of freshwater perturbation in the last 10,000 years is the ~ 160-yr cold anomaly at 8.2 ka BP^3,4^, widely attributed to a slowdown of the Atlantic Meridional Overturning Circulation (AMOC) following the input of large volumes of freshwater to the North Atlantic through Hudson Bay and Strait^5–11^. This rise in freshwater delivery to the ocean has been attributed to the drainage of glacial Lake Agassiz–Ojibway (LAO)^12–14^ which formed along the southern margin of the Laurentide Ice Sheet (LIS) during its northward retreat (Fig. 1). LAO was separated from the ocean by the Hudson Bay Ice Saddle (HBIS) that collapsed sometime between 8.6 and 8.1 ka^15,16^. Although LAO drainage undoubtedly supplied freshwater to the ocean, its role as the primary forcing mechanism for the 8.2 ka AMOC slowdown has been questioned, particularly with respect to the magnitude and duration of the freshwater pulse required to sustain the observed climate anomaly. In this context, several modelling studies propose alternative or complementary mechanisms, including a stronger contribution from background freshwater fluxes associated with ongoing LIS decay and the eventual collapse of the HBIS^17–20^. These contrasting interpretations underscore substantial uncertainty regarding the structure, magnitude, and duration of freshwater forcing during this interval^21^.

Recent work has attempted to reconstruct the chronology and mechanisms of meltwater discharges from oceanic records^22,23^ and continental archives^24^. However, the lack of consensus on the timing and sequence of events recorded in marine sedimentary archives – largely driven by inconsistent age models between cores and variability in key stratigraphic unit interpretations – remains a major limitation for resolving the relative roles of the different freshwater sources during the 8.2 ka event. For example, the classic hematite-rich “red bed,” long used as a chronostratigraphic marker for the final LAO drainage^13,23,25,26^, has been reinterpreted as reflecting the opening of Hudson Bay^22^. Moreover, the suggestion that multiple red layers may exist^22^ further complicates core-to-core stratigraphic correlations. Despite general agreement that substantial freshwater discharges (or LAO drainages) occurred more than once during the Early Holocene, the time offsets between events^16,24,27,28^ and their relative magnitudes and impact remain to be fully resolved. These problems highlight the need to better constrain the sedimentary record of Hudson Bay deglaciation and its associated freshwater forcing, to harmonize both continental records and distal marine sedimentary signals before, during, and after the 8.2 ka event. Here, we present a new high-resolution Early Holocene sedimentary sequence from two marine cores collected in western Hudson Strait (Fig. 1), a key location at the outlet of Hudson Bay, directly downstream of the LAO drainage pathway. These records allow for the establishment of a refined event chronology, correlation of stratigraphic units with other marine records, and evaluation of the roles of the HBIS collapse and LAO drainage in shaping the freshwater regime that coincided with—and may have contributed to—the 8.2 ka AMOC perturbation.

### Lithostratigraphic characterisation of the Hudson Strait cores

**Fig. 2:**
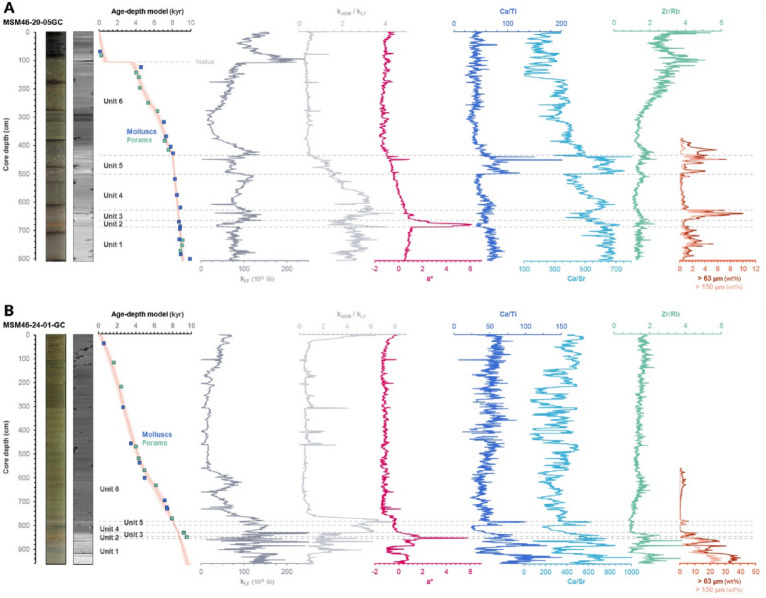
For each core, panels show core photographs, CT-scan density images, calibrated age–depth models, and downcore variations in colour (a*), magnetic susceptibility, elemental ratios (e.g., Ca/Ti, Ca/Sr), and grain-size fractions (> 63 μm and > 150 μm wt%). Stratigraphic units (Units 1–6) are indicated. (**A**). Charts for MSM46-20-05-GC. (**B**) Charts for MSM46-24-01-GC.

**Fig. 3:**
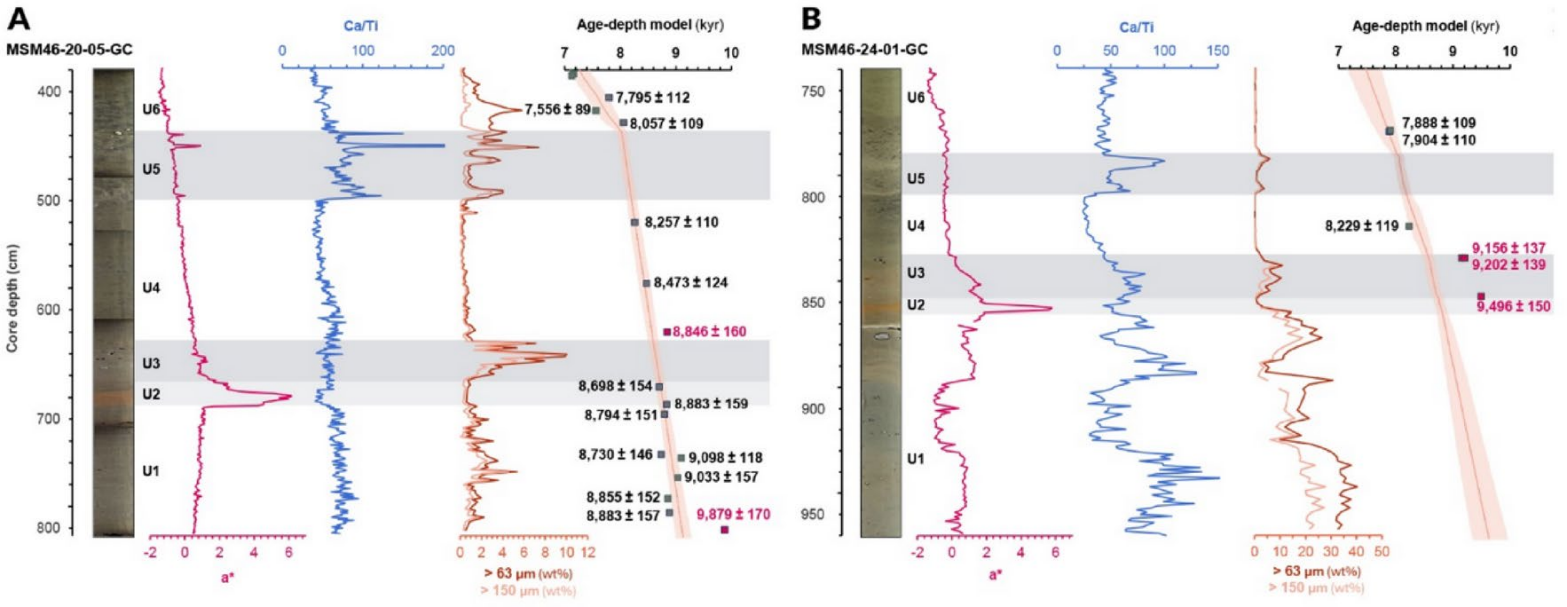
Detailed view of the stratigraphic transitions defining Units 1–6 in the Hudson Strait cores. (**A**) Core 20 (MSM46-20-05-GC). (**B**) Core 24 (MSM46-24-01-GC). For each core, panels show core photographs, a* colour index, Ca/Ti ratio, IRD abundances (> 63 μm and > 150 μm), and the age–depth model with individual calibrated radiocarbon ages. The light grey band corresponds to Unit 2. The darker grey bands correspond to Unit 3 and 5 IRD events.

Cores MSM46-20-05-GC (hereafter “core 20”) and MSM46-24-01-GC (“core 24”) record a continuous Early Holocene sedimentary sequence divided into six stratigraphic units (Units 1–6; Figs. 2 and 3) defined by colour, CT-scan density contrasts, sedimentary structures, and supported by geochemical, magnetic, and grain-size proxies. Both cores show similar unit successions, although their internal variability differs due to their relative positions along a proximal–distal gradient, with Core 24 lying closer to the former ice margin. Each unit is described below, from oldest to youngest.

Unit 1 comprises the basal sediments recovered in both cores and consists of brown to grey muds with low a* values and moderate magnetic susceptibility. Core 24 contains markedly coarser material (sand, gravel, and pebbles) producing high variability in kLF, a*, Ca/Ti, Ca/Sr, and grain-size fractions, whereas Core 20 is finer-grained and more homogeneous. Both cores show moderately elevated Ca/Ti and Ca/Sr relative to overlying Unit 2, although IRD content is substantially higher and more variable in Core 24, consistent with its closer proximity to the former ice margin. In Core 20, IRD content is lower and occurs as subdued peaks without large shifts in geochemistry or colour.

Unit 2 forms a visually distinct red bed with a sharp basal contact over Unit 1. Relative to Unit 1, Unit 2 exhibits strongly increased a* values, sharply reduced magnetic susceptibility, lower Ca/Ti, and comparatively stable but elevated Ca/Sr. IRD content drops abruptly in both cores, and CT imagery shows a homogeneous fine-grained structure without coarse layers.

Unit 3 overlies the red bed and is characterized by a return to lower a* values, moderate kLF, and generally stable Ca/Ti and Ca/Sr. Unlike Unit 2, Unit 3 contains coarse and abundant IRD, including scattered pebbles and several prominent IRD peaks concentrated in the upper portion of the unit. The increases in IRD content occur without clear accompanying changes in Ca/Ti or Ca/Sr, marking a clear physical but muted geochemical contrast with Unit 2. However, a small peak in a* values appears synchronous to the rise in IRDs. An absence of carbonate clasts is also observed in the > 63 μm fraction.

Unit 4 is comparatively uniform, with steadily declining a*, minimal IRD, and gradually decreasing Ca/Ti relative to Unit 3. Ca/Sr shows only a slight initial rise before trending downward, while magnetic susceptibility and Zr/Rb display little internal variation. The unit contains no coarse layers and appears mostly homogenous in CT images, representing a sustained interval of low-energy sedimentation.

Unit 5 is highly structured and displays the largest proxy variability in the sequence. Two prominent peaks in Ca/Ti and Ca/Sr, accompanied by synchronous rises in a* and IRD, mark its internal architecture. Relative to Unit 4, Unit 5 exhibits an abrupt increase in both coarse and fine detrital components, with IRD-rich laminae visible in CT scans. These two main peaks are reproducible in both Hudson Strait cores and in one Labrador fjord core.

Unit 6 consists of fine-grained, IRD-free muds with low a*, low and stable Ca/Ti, and generally uniform magnetic susceptibility. Ca/Sr remains stable except for moderate variability in the upper 1–2 m of Core 24; Zr/Rb shows a gentle upward increase. In Core 20, grain size shows a gradual upward coarsening between ~ 2.8 and 1.1 m depth though the unit remains otherwise homogeneous.

### Chronology and unit correlation across Hudson Strait and Labrador shelf

**Fig. 4:**
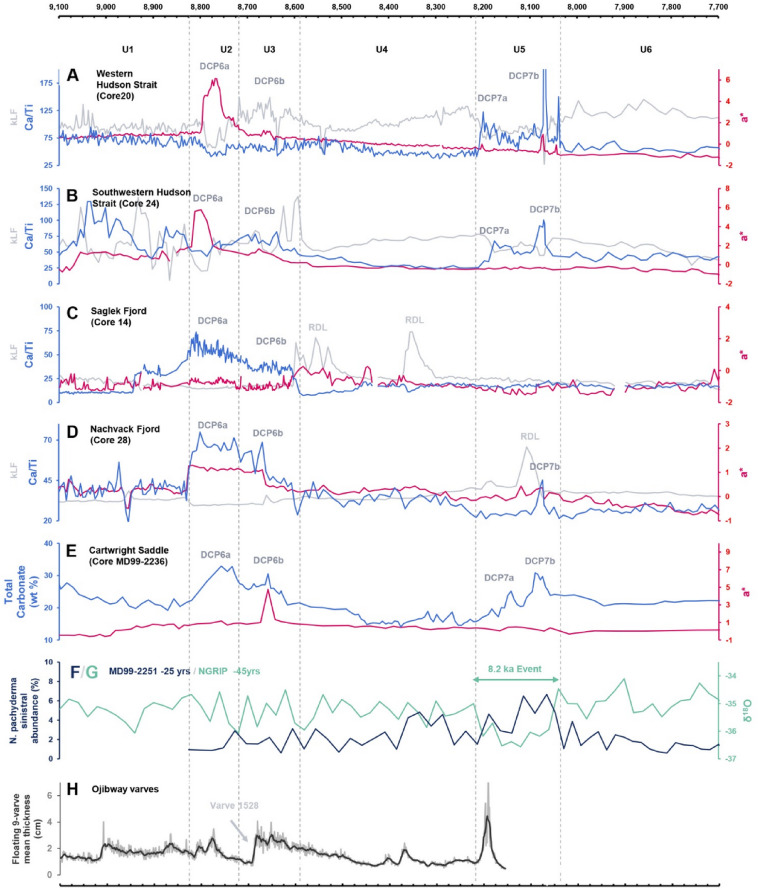
Core chronologies, inter-core correlations, and stratigraphic units compared with regional records of Early Holocene deglaciation in Hudson Bay and Hudson Strait. (**A**) Core MSM46-20-05GC (Hudson Strait) showing Ca/Ti, kLF, and a* profiles. Peaks labelled DCP6a–b and DCP7a–b are interpreted as correlative to the detrital-carbonate-rich event template defined on the Labrador Shelf in core MD99-2236^22^, based on shared proxy structure and stratigraphic position. (**B**) Core MSM46-24-01GC (Hudson Strait), displaying Ca/Ti, kLF, and a* and illustrating similar unit boundaries and DCP peak architecture to Core 20. (**C**) Core MSM46-14GC from Saglek Fjord^34^, showing Ca/Ti, kLF, and a*. Two rapidly deposited layers (RDLs), interpreted as turbidites^34^, truncate or rework the upper part of DCP6b, suggesting that the post-DCP6 chronology in this core may be younger than plotted. (**D**) Core MSM46-28GC from Nachvak Fjord^34^, displaying Ca/Ti, kLF, and a*. (**E**) Core MD99-2236 from the Labrador Shelf^22^, showing total CaCO₃ and a*. (**F**) North Atlantic core MD99-2251 record of Neogloboquadrina pachyderma (sinistral) abundance^56^, plotted alongside δ¹⁸O from the NGRIP Greenland ice core. The interval interpreted as the 8.2 ka event is highlighted on both records, permitting comparison with the timing and duration of Unit 5 detrital-carbonate peaks in the Hudson Strait sequence. (**G**) Nine-year-average varve thickness from the Ojibway varve records^41,42^, adjusted to the alternative Connaught chronology^55^.

Establishing a coherent chronology for deglaciation of Hudson Bay and Strait is complicated by spatially variable marine reservoir corrections, the influence of mixed carbonate sources on foraminifera, and the potential for reworked or contaminated radiocarbon material. Despite these challenges, the two Hudson Strait records exhibit a remarkably consistent stratigraphic architecture that can be aligned with other published sequences from Hudson Bay and Strait^13,14,25,26,33^, as well as with carbonate-rich intervals documented on the Labrador Shelf^22,23^ and in the Labrador fjords^34^ (Fig. 4). Importantly, correlation is based not on absolute proxy magnitudes—which differ due to distance from sediment sources, basin physiography, and local hydrodynamics—but on the internal structure of proxy peaks, troughs, and inflection points as well as on the relative stratigraphic architecture preserved across all records.

Across these settings, the detrital-carbonate sequence described on the Labrador Shelf^22^ provides the clearest correlative template. The upper part of their DCP succession (i.e., DCP6 and DCP7) and the intervening intervals can be subdivided into six sedimentary units whose proxy structure closely mirrors that observed in the Hudson Strait cores (Table 1). DCP6 is characterized by a dual event (DCP6a and b), with the first Ca peak being more prominent; DCP7 also comprises two distinct phases, expressed as an initial modest peak followed by a larger main peak, although not originally divided/labelled “7a” and “7b”. This structural pattern is present in the Hudson Strait records, albeit with differing magnitudes (Fig. 4). Unit 2 and 3 succession appears as a double a* maximum in Hudson Strait equivalent to DCP6a and b, with the first excursion more prominent. Unit 5 is expressed most clearly as a dual structure in Ca/Ti equivalent to DCP7a and b, with the second peak being dominant. In other words, the total carbonate proxy in MD99-2236 can be partitioned into the same six subdivision intervals recognised in our Hudson Strait sequence.

Equivalent structures are visible in the Ca/Sr and Ca/Ti records of the Labrador fjords^34^ and the Labrador Shelf^23^ with distinctive peaks that can be correlative to DCP6 and 7 (Fig. 4). Although the second event is not as clearly expressed in the fjord sequences—and in at least one case may have been partially removed by rapidly-deposited, erosive turbidites—a Ca/Ti and Ca/Sr peak at the appropriate stratigraphic level in both fjord record nonetheless provides a plausible correlative to DCP7b. MSM-19-2 collected in the Saglek Basin on the shelf, just out of Saglek Fjord, displays a similar proxy structure with a carbonate event expressed as peaks in Ca/Sr and a* correlative to DCP6 and a Ca and a* peak probably correlative of DCP7b.

Based on these correspondences, the Labrador Shelf/fjord proxy template can be correlated to the Hudson Strait record as follows: sediment preceding DCP6a corresponds to Unit 1; DCP6a to Unit 2; DCP6b to Unit 3; the interval between DCP6b and the onset of the next dual event to Unit 4; DCP7a and b to Unit 5; and all sediment above the termination of DCP7b to Unit 6. These relationships confirm that the internal architecture of our six-unit model reflects the same basin-wide deglacial pattern expressed across the entire Hudson Bay–Strait and Labrador system, even though the expressions are modulated by distance from source areas and local sediment routing.

Using these regionally traceable stratigraphic markers as tie-points, and integrating them with weighted-mean calibrated radiocarbon ages and composite Bayesian age–depth modelling, a coherent chronology can be assigned to each unit boundary (Table 1; Fig. 4). Collectively, Units 2–5 span roughly 780–860 years between ~ 8.80 and 8.01 ka, with individual units ranging from ~ 70–430 years in duration.

The overall coherence in stratigraphy, proxy structure, and calibrated boundary ages across Hudson Bay and Strait, the Labrador Shelf, and the Labrador fjords supports the interpretation that Units 1–6 represent a regionally synchronous sequence of deglacial events. This integrated temporal framework provides the foundation for reconstructing ice-margin dynamics, freshwater forcing, and sedimentary processes in the Hudson Bay–Strait system during the interval leading up to and following the 8.2 ka event.

**Table 1:** Age model for the major stratigraphic units of the Hudson Bay, Hudson Strait and Larabdor shelf and fjords, and correlative detrital carbonate peaks from the Labrador Sea.

Unit	DCP^22^ (Jennings et al., 2015)	Base age (yr BP)	Top age (yr BP)	Duration (yrs)
1	–	Core 20 = 9,131 ± 123 year BP; Core 24 = 9,699 ± 305 year BP	8,823 ± 44	Core 20 = 346 yrs Core 24 = 875 yrs
2	6a	8,823 ± 44	8,718 ± 49	105 ± 63 yr
3	6b	8,718 ± 49	8,588 ± 46	130 ± 64 yr
4	–	8,588 ± 46	8,181 ± 49	407 ± 67 yr
5	7	8,181 ± 49	8,053 ± 45	128 ± 67 yr
6	–	8,053 ± 45	core tops	8,050 ± 45 yr

### Depositional processes and sedimentary model

The proxy records of the Hudson Strait cores define six successive units (Units 1–6) that we interpret in terms of changing depositional regimes, evolving ice–ocean configuration, and shifts in sediment provenance through the final collapse of the LIS over Hudson Bay. Below we outline the dominant processes consistent with the proxy systematics, while highlighting uncertainties, alternative mechanisms, and parallels with previous work from Hudson Bay and Strait and the Labrador margin.

**Fig. 5:**
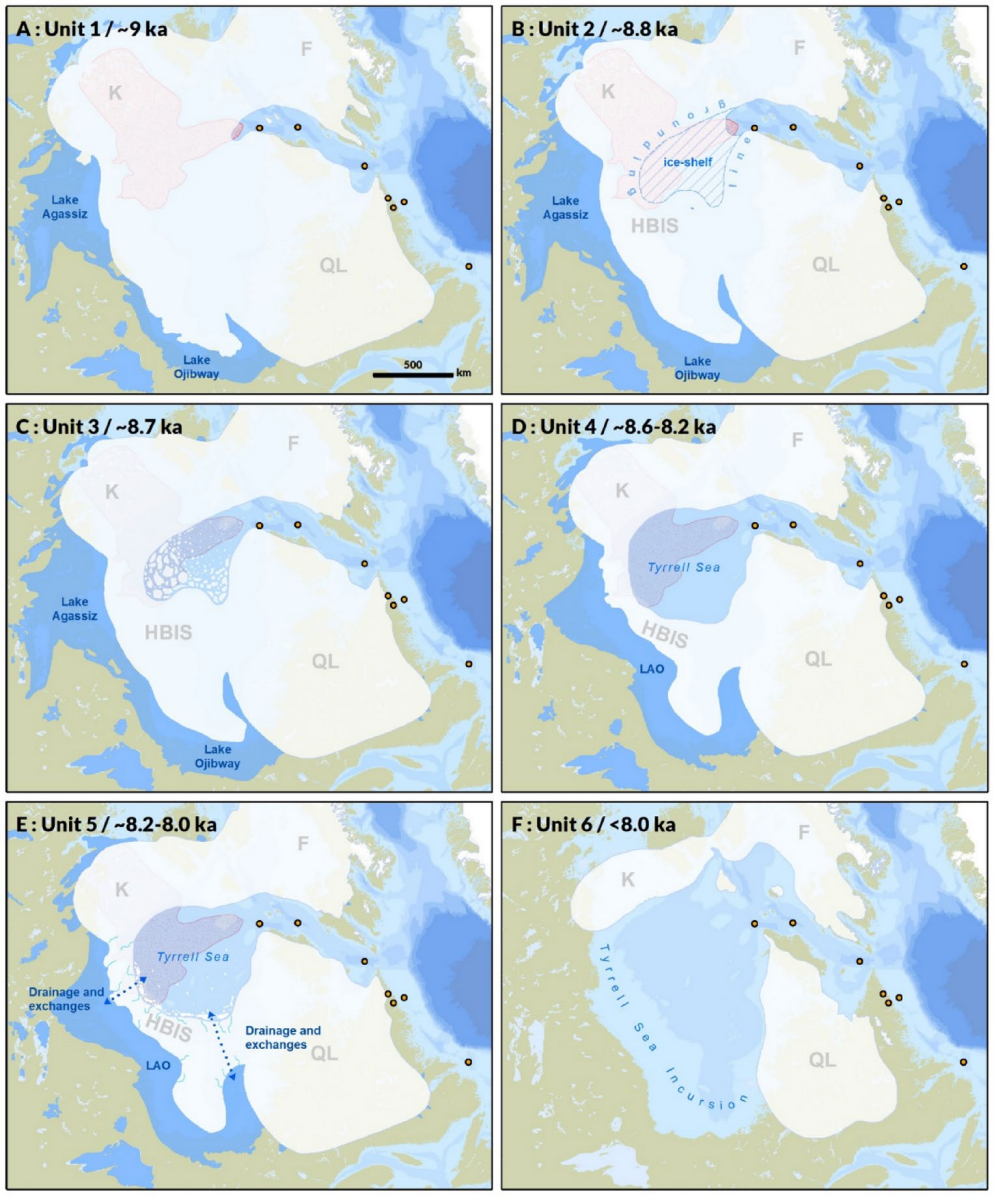
Paleogeographic reconstructions of Hudson Bay deglaciation inferred from the stratigraphic interpretation of Hudson Strait sedimentary units. Ice domes of the Laurentide Ice Sheet are indicated as K (Keewatin), QL (Québec–Labrador), and F (Foxe). (**A**) Reconstruction at 9.0 ka, representing conditions during deposition of Unit 1. Ice and lake extents from^29^ (8.9 ka). Paleotopography and bathymetry from the ICE-7G model at 9.0 ka^46^. (**B**) Reconstruction at ~ 8.8 ka showing grounding-line retreat and formation of an ice shelf over the Dubawnt dispersal train (modified from^30,31^), corresponding to deposition of Unit 2. Ice and lake extents modified from^29^ (8.6 ka). Paleotopography and bathymetry from the ICE-7G model at 9.0 ka^46^. (**C**) Reconstruction at ~ 8.7 ka illustrating ice-shelf breakup and renewed iceberg discharge, representing conditions during deposition of Unit 3. Ice and lake extents modified from^29^ (8.6 ka). Paleotopography and bathymetry from the ICE-7G model at 8.5 ka^46^. (**D**) Reconstruction spanning ~ 8.6–8.2 ka, representing conditions during deposition of Unit 4, characterized by stabilized ice margins and reduced iceberg flux. Lake extents modified from^29^ (8.5 ka) and ice extents from^29^ (8.5 ka). Paleotopography and bathymetry from the ICE-7G model at 8.5 ka^46^. **E**. Reconstruction at ~ 8.2 ka showing breakup of the Hudson Bay Ice Saddle (HBIS) and drainage of glacial Lake Agassiz–Ojibway, with establishment of marine–lacustrine exchanges, corresponding to deposition of Unit 5. Ice and lake extents modified from^29^ (8.5 ka) and^57^ (8.5 ka). Paleotopography and bathymetry from the ICE-7G model at 8.0 ka^46^. **F.** Reconstruction at ~ 8.0 ka illustrating post-collapse conditions following complete drainage of Lake Agassiz–Ojibway and loss of the HBIS, corresponding to deposition of Unit 6. Ice extents from^57^ (8.0 ka). Paleotopography and bathymetry from the ICE-7G model at 8.0 ka^46^.

Unit 1 is characterised by brown to grey muds with moderately elevated Ca/Ti, Ca/Sr, and a*, and variable IRD content, especially in the coarser, more heterogeneous sediments of Core 24. The presence of reddish detritus and moderate Ca/Ti and Ca/Sr values indicates incorporation of a mix of carbonate-rich and reddish material, consistent with the characteristics of tills described on nearby islands^35–37^ and on the Hudson Bay seafloor^31,38^. The more heterogeneous (alternating low and high kLF values) and IRD-rich character of Core 24 relative to Core 20 suggests a proximal–distal gradient in iceberg calving intensity and/or grounding-line oscillations, with Core 24 lying closer to the former ice margin. Variations in kLF in core 24 are not synchronous with changes in other sedimentological or geochemical proxies and likely reflect highly variable terrigenous inputs from nearby ice margins bordering the strait. Specifically, low kLF values likely reflect episodic dilution of magnetic minerals by fine-grained siliciclastic or carbonate-rich sediment supplied by proximal ice margins during the early opening of Hudson Bay, before the influence of the red dispersal train is detected, as indicated by the absence of a corresponding change in a* values. Sedimentation during Unit 1 was likely controlled by a characteristic set of glaciomarine processes^39^: meltwater-driven suspension plumes transporting moderate detrital carbonate and red fines, tidewater discharge of debris-rich ice and attendant IRD fallout, and local remobilisation of subglacial or ice-marginal sediment by bottom currents (Fig. 5a). The absence of sharp internal geochemical breaks within the unit suggests that no major reorganisation of meltwater routing or sediment sources occurred during its deposition.

Unit 2 is defined by a sharp increase in red coloration (a*), a marked decrease in magnetic susceptibility and Ca/Ti, and strongly muted IRD delivery in Hudson Strait cores. Although Ca/Ti decreases in Hudson Strait, the correlative horizon on the Labrador Shelf and in fjords is marked by a prominent Ca/Ti or bulk Ca peak^22,23,34^. This contrast is best explained by proximity to sediment sources and differential settling behaviour of terrigenous (Ti-rich) and carbonate (Ca-rich) particles: Ti-rich clastics settle rapidly near the grounding line, masking carbonate signatures in proximal settings (Hudson Bay/Strait), whereas finer carbonate-bearing silts and clays remain in suspension and are preferentially exported to distal basins (Labrador Shelf/fjords). As a result, the Labrador records capture the carbonate component of this event more clearly, while the Hudson Strait expression has a more prononced terrigenous signature. The traditional interpretation of this “red layer” links it to intense erosion and redistribution of Dubawnt-derived, hematite-rich tills from western Hudson Bay^13,22,23,25,33,34^. Our Hudson Strait records support this origin: the steep rise in a* and concomitant decrease in kLF can be attributed to the high hematite content of fines sourced from Dubawnt red tills. Unit 2 therefore reflects a short-lived but regionally traceable episode of enhanced erosion of both (i) Dubawnt tills and (ii) the Paleozoic carbonate platform or carbonate-rich tills beneath western Hudson Bay, accompanied by strongly suppressed IRD. This combination requires a short-lived but fundamental change in sediment source, routing, and/or basal conditions that does not involve substantial iceberg production. Previous interpretations have linked the red bed to the drainage of LAO, either through catastrophic failure of the HBIS/LIS ice dam^13,33^ or through a series of subglacial drainages beneath the ice dam^14^, both scenarios most likely involving intense iceberg production. It is ambiguous if a partial lake drawndown through subglacial drainages has to involve iceberg production, but it would provide a natural mechanism for concentrated erosion and transport of red sediment via high-energy, pressurised flows^40^; in this context the IRD-rich layer of Unit 3 could be interpreted as an iceberg response to dam destabilisation, consistent with arcuate iceberg scours mapped on the Hudson Bay seafloor^14,38^. However, an early subglacial lake drawdown at ~ 8.8 ka does not align with any clear discontinuity in the varve records of Lake Ojibway^41,42^ nor with currently available lake-level reconstructions^43,44^. The only change in the Ojibway varve sequence that could fit with the timing of the red bed is the pronounced varve thickening at varve 1528 (Fig. 4h), but was interpreted as a major hydrological shift likely associated with the coalescence of LAO^41^. These considerations favours an alternative mechanism, in line with recent studies that attribute the red bed primarily to the early deglaciation and opening of Hudson Bay^22,23^, rather than to the final LAO drainage. However, large-scale deglaciation of Hudson Bay would normally be expected to generate extensive iceberg production. Reconciling the strong fine-fraction signal of Unit 2 with its muted IRD therefore requires an additional constraint on ice geometry. We propose that the most consistent mechanism is a rapid episode of grounding-line retreat and partial ungrounding of the LIS margin over western Hudson Bay, forming a buttressed ice shelf and allowing marine waters to intrude beneath the ice front. Palaeobathymetric reconstructions^45,46^ indicate that glacio-isostatic depression created a reverse bed slope up to ~ 200–225 m deeper than present, reaching over 400-m paleo-depths^46^. This context strongly favoured rapid inland penetration of seawater along retrograde beds, analogous to marine-terminating glaciers and ice shelves in Greenland and Antarctica today^47–49^. The strongly muted IRD in Unit 2 is consistent with partial ice-shelf buttressing: a floating or near-floating margin reduces basal traction, limits grounding-zone stress transmission, and suppresses coarse-debris entrainment, even while sub-ice oceanic current circulation efficiently entrains and exports fine sediment. In this perspective, Unit 2 (8,823 ± 44 to 8,718 ± 49 year BP; duration ≈ 105 ± 63 year) records a brief ungrounding and marine-intrusion phase that reorganised sediment routing and basal conditions across western Hudson Bay, setting the stage for subsequent destabilisation (Fig. 5b).

From Unit 2 to Unit 3, the most significant change is the return to higher > 63 μm and > 150 μm fractions, marked by IRD-rich layers but with an absence of carbonate clasts (Fig. 3). Ca/Ti and Ca/Sr both show modest decreases but retain a small peak that is correlative to DCP6b on the Labrador Shelf (Fig. 4); these shifts are accompanied by minor increases in a* and kLF. The lack of a strong Ca enrichment, together with the absence of carbonate clasts in this IRD-dominated interval, indicates a decoupling between coarse debris delivery and carbonate content. This decoupling suggests that Unit 3 deposition was controlled mainly by mechanical release of sediment that was already englacial or intra-shelf, rather than by renewed erosion of the Paleozoic carbonate platform or carbonate-rich tills on the Hudson Bay seafloor. An additional compatible mechanism is that iceberg sources during Unit 3 were routed preferentially from the margins of western and eastern Hudson Bay, where palaeo-ice flow into the Tyrrell Sea intersected more restricted Paleozoic carbonate platforms than in the south and central basin^50^. Meltwater and icebergs from the northwestern margin of the Labrador Dome could therefore also have contributed to IRD delivery with limited carbonate content. In this scenario, the subdued carbonate signature reflects changes in calving-front geometry and source regions rather than a fundamental shift in subglacial erosion processes. We therefore interpret Unit 3 as reflecting enhanced calving and melt-out of debris already entrained within floating or near-floating ice, which is consistent with ice-shelf thinning and mechanical disintegration following the ungrounding/grounding-line retreat event represented by Unit 2 (Fig. 5c). This interval, which began at 8,718 ± 49 year BP and lasted for ~ 130 ± 64 year, is best understood as a rapid iceberg-release phase associated with continued margin retreat and widening of the Tyrrell Sea, rather than the collapse of the HBIS itself. Minor grounding-line oscillations, limited till reworking, or local debris flows may also have occurred throughout the deposition of this unit. However, the overall downward trend in Ca/Sr and Ca/Ti (aside from the modest DCP6b peak), the short duration of the unit, and the renewed IRD flux without evidence for widespread carbonate excavation, collectively favour an interpretation involving ice-shelf breakup and ice margin readjustment.

The transition from Unit 3 to Unit 4 is marked by renewed reduction in IRD input, coupled with progressively decreasing Ca/Ti and a* values and minimal internal variability in all magnetic and geochemical proxies. Together, these trends indicate a shift toward lower-energy glacimarine sedimentation. Such conditions may reflect a more stable ice margin, reduced iceberg production, and/or increasing distance of our cores to major meltwater and sediment sources as the LIS margin likely continued to recede and underwent thinning. The very subtle internal changes in Ca/Sr and Ca/Ti likely represent gradual adjustments in sediment provenance associated with progressive retreat, rather than discrete depositional or hydrological events. CT imagery and grain-size distributions show no evidence of high-amplitude turbidity, meltwater pulses, or coarse-grained IRD events, supporting interpretation of Unit 4 as a background interval separating the more dynamic depositional phases represented by Units 3 and 5, spanning approximately ~ 407 ± 67 years from 8,588 ± 56 ka to 8.181 ± 49 ka (Fig. 5d).

 Unit 5 differs sharply from Unit 4 across several proxies. Magnetic susceptibility drops markedly, while Ca/Ti and Ca/Sr develop two prominent high-amplitude peaks; IRD spikes occur in both cores, and in Core 20 the a* record rises synchronously with Ca and IRD. These peaks are the most pronounced Ca/Ti and Ca/Sr peaks in the entire sequence and represent the last major detrital-carbonate event, as subsequent variations in Unit 6 do not show any comparable shift in sedimentation. Similar dual-peaked carbonate–IRD structures have been reported in records from the Labrador Shelf^22^ and in other Hudson Strait cores^14,26^ and imply the final series of iceberg-production pulses and enhanced detrital carbonate erosion and delivery through Hudson Strait during the Early Holocene. Mechanisms consistent with these proxies include destabilisation of the marine-terminating LIS margin in Hudson Bay — during either retreat or short-lived readvances — and outburst floods associated with LAO drainage. Most importantly is the timing of the deposition of Unit 5 — from 8,181 ± 49 to 8,053 ± 45 yrs BP — that overlaps with the best current estimates for the final HBIS collapse (8.1 ka BP)^16,51,^ thetwo-stage drainage of LAO (8.3–8.1 ka BP)^22,24,51,52^ and the onset of sustained exchanges between marine and glaciolacustrine waters, leading to Ojibway varves containing marine fauna in the James Bay region (8.1 ka)^52,53^. The duration (128 ± 67 year) and structure of the proxies are also consistent with multiple LAO drainage events seperated by ≥ 65 year (Connaught varve sequence duration)^41,42,54,55^. Overall, both LIS destabilisation and multiphase LAO drainage could plausibly contributed to erosion and transport of carbonate rich material and produce the paired IRD–carbonate peaks seen in our cores, but limitations in resolving the precise cause–effect structure require a cautious interpretation. We therefore consider Unit 5 as a composite interval of intensified iceberg discharge (iceberg scours)^14^ and detrital carbonate export. Its two dominant peaks most likely reflect regionally significant, meltwater-driven destabilisation linked to the final disintegration of the LIS over Hudson Bay, failure of the HBIS dam, and basin-wide erosion and flushing of fine carbonate-rich sediment during the final drainage of LAO (Fig. 5e).

The transition from Unit 5 to Unit 6 is recorded by a marked reduction in the magnitude of proxy variability, including muted colour, stable Ca/Ti, and subdued Ca/Sr, beginning at 8,053 ± 45 year BP. These characteristics mirror early postglacial intervals elsewhere in the region and are consistent with the establishment of fully open-marine conditions^22,26,34^. Unit 6 thus reflects postglacial hemipelagic sedimentation with no evidence for large-scale changes in sedimentation that could be associated with major iceberg rafting, meltwater pulses, or significant ice-margin oscillations. Subtle upward changes in Ca/Sr and grain size in Core 24 may record minor variations in sediment transport efficiency or sediment focusing, but they do not indicate enhanced meltwater flux or renewed coarse debris contributions. Overall, Unit 6 documents ~ 8 kyr of relatively stable, open-marine sedimentation following the final collapse of the LIS over Hudson Bay (Fig. 5f).

### Freshwater regimes and climate implications

The stratigraphic architecture and proxy structure of Units 1–6 in the Hudson Strait cores, when integrated with correlative sequences from the Labrador Shelf and fjords, reveal a multi-phase evolution of freshwater delivery from the Hudson Bay sector during the Early Holocene. Rather than a uniform freshwater flux through time, the Hudson Strait record captures a series of reorganisations in ice-sheet geometry, basal hydrology, sediment routing, and ocean–ice interactions; each involving with distinct freshwater-forcing modes relevant to the sensitivity of North Atlantic overturning. A central outcome of our record is that, while geochemical amplitudes vary across basins due to distance from source, sediment availability, and hydrodynamic sorting, the internal sediment architecture of events – including peak symmetry, dual structures, and their association with IRD pulses – remains remarkably consistent. This synchronicity indicates that the depositional units reflect regionally coherent freshwater and sediment-export phases, and that the Hudson Strait cores capture the proximal manifestation of freshwater forcing that ultimately influenced the state of the subpolar gyre and AMOC. These phases collectively frame the freshwater forcing that ultimately led into, and shaped, the 8.2 ka climatic event.

The first freshwater phases corresponds broadly to Unit 1 and reflects background deglacial meltwater and iceberg discharge preceding ~ 8.8 ka. Proxy evidence indicates that Hudson Strait experienced steady, moderate inputs of terrigenous and carbonate-rich fines, along with variable IRD delivery, particularly in the southwestern core (Core 24). The weak IRD signal in Core 20 suggests that icebergs calved into Hudson Bay and the Tyrrell Sea did not consistently transit into the eastern strait, implying limited long-distance iceberg transport and that freshwater released through calving was mostly confined to the interior basins. Freshwater reaching Hudson Strait in this interval was most likely supplied by surface runoff and ablation of the nearby Quebec–Labrador Ice Dome, with potential contributions from the Foxe Dome, as well as diffuse export of brackish Tyrrell Sea surface waters. This configuration is consistent with climate-model simulations indicating that before ~ 8.6–8.5 ka the North Atlantic experienced gradually increasing freshwater input derived mainly from ongoing decay of continental ice masses rather than from discrete outburst event^17,19^. In this framework, Unit 1 may be considered as a preconditioning phase during which freshening of the Labrador Sea was modest but persistent^21^, delivered through broadly distributed pathways and without major reorganisations of the Hudson Bay–Hudson Strait system.

A fundamentally different freshwater mode emerges with the closely linked events represented by Units 2 and 3. These units reflect a short-lived perturbation to ice–ocean geometry in western Hudson Bay, initiated when marine waters intruded beneath the ice along a glacio-isostatically deepened reverse-slope bed. Rapid ungrounding in this context likely promoted the formation of an ice shelf and sharply increased sub-ice-shelf/basal melt; a mechanism that has been under scrutinity in modern environment for some time^58–62^ and for which models show that the attendant outcomes implicate major changes in ice-sheet dynamics and mass balance^63,64^. This expansion of the ice–ocean interface, together with strong circulation beneath the newly formed shelf, likely facilitated the entrainment and export of fine-grained sediments that produced the red bed of Unit 2. IRD delivery was suppressed because the ice shelf was buttressed and basal traction reduced, which is consistent with the near absence of coarse debris during this interval. However, since the grounding line lay on a retrograde bedrock slope well below sea level, the interior of the LIS was likely prone to the marine ice-sheet instability mechanism^65,68^. Accordingly, continued sub-ice-shelf melt subsequently thinned the shelf to the point of mechanical failure and break-up, producing the IRD-rich deposit of Unit 3. This break-up would have triggered a transient increase in open-water area within Hudson Bay and, consequently, a reduction in local albedo. This enhanced absorption of solar energy, espacilly during seasonally ice-free Hudson Bay, would have amplified local ablation and margin retreat until a new short-lived equilibrium was established^69^. Once this geometry stabilised, the greatly expanded ice–ocean contact across Hudson Bay most likely provided enhanced background freshwater delivery to the ocean during Unit 4 (8.6–8.2 ka). This increase in freshwater inputs to the North Atlantic during Unit 4 is consistent with the pulsed acceleration of global sea-level rise starting around 8.5–8.4 ka in various intermediate and far-field relative sea-level records^70–75^. The return to low-energy sedimentation in Unit 4 aligns with Early Holocene climate simulations that predict a gradual cooling trend after ~ 8.6 ka, which is consistent with the establishment of a more stable glacimarine environment sensitive to relatively modest variations in freshwater flux^11,76^.

The final and climatically most consequential freshwater mode corresponds to Unit 5. This interval contains major terrigenous-carbonate peaks and IRD pulses. It has a duration and timing that is statistically indistinguishable from the length of the 8.2 ka δ¹⁸O anomaly recorded in Greenland ice cores^3,4^. The synchronicity between these records, the dual-peaked detrital-carbonate events in Hudson Strait and on the Labrador Shelf, as well as independent chronologies for the final drainage phases of LAO strongly indicate that Unit 5 captures a multi-stage freshwater release into the Labrador Sea. While the final LAO drainage clearly forms a central component of this interval, the broader paleogeographic configuration of the waning LIS also likely played a critical role. Prior to ~ 8.2 ka, meltwater from the retreating Keewatin and Québec–Labrador domes was routed largely toward the North Atlantic through the St. Lawrence system or temporarily stored within proglacial lakes. Following the collapse of the Hudson Bay ice saddle, this drainage was abruptly redirected toward Hudson Bay, establishing a new, far more direct pathway for continental freshwater export into the Labrador Sea (Fig. 5). AMOC is believed to be highly sensitive to the location of freshwater forcings^8,21,77^. Therefore, this large-scale reorganisation of meltwater routing combined with the multiphase LAO outburst likely amplified both the duration and the climatic impact of the freshwater forcing associated with the 8.2 ka event^7,18,21^^78^.

A further consideration is that Unit 5 may record positive cryosphere–ocean feedbacks. The initial freshwater release associated with lake drainage and/or saddle collapse would have cooled the North Atlantic surface layer, expanded sea-ice cover, increased albedo, and promoted cooler temperatures, which in turn created favorable contiditions for stabilization and readvance of LIS margin. This interpretation is supported by the widespread occurrence of moraine complexes dated at ~ 8.2 ka—including the Sakami moraine (Québec)^69,79,80^, Sebaskachu (Labrador)^81^, Cockburn moraines (Baffin Island)^82–84^, the Chantrey and MacAlpine systems (Keewatin)^15,82^ and correlative advances in Greenland^85–87^— which all point to a near-synchronous, circum-Labrador/Baffin Bay ice-margin response to regional cooling. Within this framework, the transient increases in iceberg discharge recorded in Unit 5 need not reflect only the mechanical consequences of meltwater drainage and routing; they may rather represent dynamic adjustments of ice margins to new ocean–atmosphere conditions. The persistence of elevated IRD flux and sustained detrital-carbonate export over ~ 130 years supports this coupled response. The stratigraphic signature of Unit 5 therefore captures not only the LAO drainage pulses but also the feedback-driven ice-margin behaviour that helped maintain freshwater forcing over multidecadal timescales. This prolonged but uneven freshwater delivery aligns with climate-model studies that argue the AMOC is particularly sensitive to sustained moderate perturbations, when background meltwater fluxes are already high and deepwater formation regions are preconditioned for destabilization^11,21,76^. Collectively, these results highlight that the sensitivity of the AMOC to Hudson Bay forcing depends not only on the volume of freshwater released, but on its duration, delivery mechanisms, and integration with changing ice geometries. Although these results do not allow quantifying freshwater fluxes or direct inference of their impact on AMOC strength, our stratigraphic framework along with recent modelling studies emphasizes the need for improved chronological, as well as volumetric constraints to disentangle the relative importance of lake-outburst, ice-sheet melting, and drainage reorganisation during this interval.

Taken together, the Hudson Strait sediment record documents a progression from a diffuse background freshwater delivery to an ocean-forced episode of rapid ice-shelf retreat and iceberg discharge, and to a final multi-stage freshwater pulse associated with lake drainage and ice-margin reorganisation. This sequence highlights that Early Holocene freshwater forcing was temporally structured, spatially variable, and governed by evolving ice–ocean geometries rather than a single mechanism. It also underlines the need for high-resolution chronologies, improved reservoir-age constraints, expanded regional tie-point networks, and additional provenance tracers to refine reconstructions of freshwater pathways and evaluate the conditions under which such events can disrupt North Atlantic overturning circulation.

## Materials and methods

### Sampling

Two sediment cores were collected during the RV *Maria S. Merian* expedition MSM46 in 2015 from two basins in western Hudson Strait (Fig. 1). The 8.07 m-long core MSM46-20-05-GC was collected in the Western Basin (63.0495 N; 74.3112 W; 426 m water depth); the 9.62 m-long core MSM46-24-01-GC was taken farther west in the Southwestern Basin, near the entrance of Hudson Bay (62.7650 N; 79.0043 W; 394 m water depth). The cores were sectioned into 1-m intervals, sealed, and stored at 4 °C for subsequent description, sampling, and analysis^88^.

### Physical properties analysis

Digital X-ray images of cores 20 and 24 were obtained from U-channels using a CT-scanner at the Institut National de la Recherche Scientifique, Centre Eau, Terre, Environnement (INRS-ETE) in Québec City. These images were used to describe lithofacies and identify intervals of coarse-debris concentrations. Multiple physical and geochemical parameters were measured at 1-cm intervals on the split cores with a Multi Sensor Core Logger (MSCL) at the Institut des sciences de la mer, Université du Québec à Rimouski (ISMER, UQAR)^89^. These include the low-field magnetic susceptibility (kLF), CIE a* colourimetry, and major and trace elemental concentrations.

Low-field magnetic susceptibility (kLF) was determined with a Bartington point sensor. This value is here used to track variations in the concentration and grain size of ferrimagnetic minerals and serves as a qualitative indicator of terrigenous inputs and sediment provenance, where lower susceptibilities reflect finer-grained hematite- or carbonate-rich material and higher values indicate enhanced influx of coarser or magnetically stronger clastic sediment^90–92^.

The a* colour component was measured with a Minolta CM-2600d spectrophotometer to quantify variations along the green–to–red spectrum. These colour changes are used as a proxy for variations in sediment provenance, particularly because positive excursions in a* have been associated with hematite-rich intervals, including the red layer previously documented in Hudson Bay and Strait^13,25,26^ and on the Labrador Shelf^22,23^.

Concentrations of Ca, Ti, Sr, Zr, and Rb were measured using an Innov-X Olympus Delta Element XRF spectrometer. XRF-derived Ca/Ti and Ca/Sr ratios are used as proxies for detrital carbonate content. Detrital carbonate peaks have been applied in numerous cores from Hudson Strait and the Labrador Shelf as markers of deglacial sediment pulses sourced from Paleozoic carbonate seafloor^22^. Zr/Rb ratios assess variations in terrigenous sediment input and relative grain size, where higher Zr/Rb indicates greater delivery of coarser material (e.g., IRD or turbidity flows), and lower ratios correspond to finer deposition^93–95^.

Anhysteretic remanent magnetization (ARM) was imparted to the U-channels by applying a 100 mT peak alternating field with a superimposed 0.05 mT DC biasing field, and measured with a 2G SRM-755 u-channel cryogenic magnetometer. The ratio kARM/kLF is an inverse indicator of magnetic grain size in the ~ 1–10 μm range, allowing assessment of subtle changes in sediment texture independent of provenance^92,96^.

The lower halves of both cores were wet-sieved at 63 μm and 150 μm to quantify coarse fractions and for evaluating the content in IRD. The abundance of sand-sized grains, granules, and pebbles embedded within otherwise fine-grained matrices was used as an indicator of iceberg rafting and episodic debris delivery, consistent with periods of enhanced calving or unstable ice-margin behaviour^97,98^.

### Chronology and age model construction

Mixed benthic foraminifera and mollusc fragments were dated from discrete intervals for establishing robust age models for both Hudson Strait cores. Forty-eight radiocarbon measurements were obtained—28 from core MSM46-20-05-GC and 20 from core MSM46-24-01-GC—at four laboratories (Leibniz–Kiel, Poznań, UCLA, and ETH Zürich; Table 2; Fig. 3). All ^14^C ages were calibrated with CALIB 8.2^99^ using the Marine20 calibration curve^100^ and a regional ΔR of − 73 ± 64 yr^101^. Although several studies applied time-varying reservoir corrections to account for enhanced sea-ice cover or the influence of ¹⁴C-depleted glacial meltwater^23,102–105^, past ΔR variability remains poorly constrained. We therefore adopt a conservative approach and apply a constant reservoir correction, following previous work in the region^22,24,106,107^. Several basal ages were rejected during model construction because they are anomalously old. These outliers likely reflect sediment reworking linked to the proximity of an ice margin, incorporation of “old” carbon from ¹⁴C-depleted meltwater, or enhanced sea-ice conditions^23^, all of which could contribute to age overestimation. Excluding these values prevents biasing the age-model toward artificially old basal ages.

Because ¹⁴C uncertainties, reservoir variability, and differing calibration approaches complicate direct inter-core alignment, we recalibrated key regional records from Hudson Strait (930023-045)^33^, the Labrador Shelf (MD99-2236^22^; MSM45-19-2^23^), and the Labrador fjords (MSM46-28-04 and MSM46-14-05)^34^ using the same calibration parameters (Marine20, ΔR = − 2 ± 69 year)^24^, resulting in a fully consistent chronological foundation for all datasets.

A first-pass Bayesian age–depth model for each core was generated using the Bacon R package^108^, and the resulting model outputs were compared at stratigraphic tie-points that are regionally traceable. Tie-points include the basal onset, peak, and termination of detrital-carbonate layers^22^, as well as reproducible inflection points in magnetic susceptibility and the visually identifiable onset and top of the red bed (Supp. Data). These markers are present in Hudson Strait records as shifts in a*, kLF, and Ca/Ti and, in Labrador Shelf and fjord cores, as synchronous detrital-carbonate peaks^22,23,34^.

Weighted-mean ages were calculated for seven regional tie-points and used as fixed chronological anchors. This approach allows reconciling minor differences in sedimentation rates and small inter-core reservoir offsets, enabling direct comparison among Hudson Strait, Labrador Shelf, and fjord records along the inferred drainage pathway of LAO (Fig. 1). Based on these tie-point constraints, final second-pass Bayesian age–depth models for all cores were produced using Bacon (Supp. Data).

By integrating calibrated radiocarbon ages, regionally harmonised calibration procedures, weighted-mean tie-points, and Bayesian age modelling, we obtained an internally consistent chronology for all cores. This framework provides the temporal basis for reconstructing the sequence, duration, and synchronicity of deglacial events across the Hudson Bay–Strait system, and constitutes the foundation for interpretation of sedimentary processes and freshwater forcing discussed above.

**Table 2:** List of AMS radiocarbon ages analyzed and calibrated in Calib8.2.

Core	Depth (m)	Sample ID	Material	Mean age (^14^C yr BP)	Uncertainty (^14^C yr BP)	Mean age (cal yr BP)	Median age (cal yr BP)	1σ-uncertainty (cal yr BP)	2σ-uncertainty (cal yr BP)
MSM46-20-05-GC	2	UCIAMS-235,858	Mollusc shell	–	–	–	–	–	–
	71	ETH-102,154	N. labradorica	580	50	90	129	90	145
	85	UCIAMS-235,859	Mollusc shells	705	15	228	234	102	196
	126	ETH-102,155	N. labradorica	4470	60	4,551	4,552	139	265
	145	KIA-54,310	Mollusc shell	4090	30	4,036	4,046	123	245
	161	UCIAMS-235,860	Mollusc shells	4305	15	4,327	4,330	116	233
	199	UCIAMS-235,861	Mollusc shells	4370	15	4,410	4,418	117	245
	251	UCIAMS-235,865	Mollusc shells	5075	15	5,329	5,314	109	228
	280	KIA-54,311	Mollusc shell	6045	30	6,354	6,351	98	206
	319	ETH-87,308	Mixed benthic foraminifera	6670	60	7,046	7,048	122	240
	369	ETH-87,309	Mixed benthic foraminifera	6895	60	7,285	7,282	106	220
	383	Poz-110,849	Mollusc shell	6770	40	7,154	7,156	108	215
	385	KIA-54,312	Mollusc shell	6735	35	7,123	7,117	108	211
	405	ETH-107,320	Mixed benthic foraminifera	7445	70	7,797	7,795	112	217
	417	Poz-110,851	Mollusc shell	7195	35	7,556	7,555	89	184
	428	ETH-87,310	Mixed benthic foraminifera	7700	60	8,057	8,059	109	226
	519	ETH-87,311	Mixed benthic foraminifera	7885	60	8,257	8,247	110	224
	575	ETH-107,321	Mixed benthic foraminifera	8095	70	8,473	8,481	124	277
	619	ETH-107,322	Mixed benthic foraminifera	8390	80	8,846	8,855	160	319
	669	ETH-87,312	Mixed benthic foraminifera	8265	70	8,698	8,698	154	279
	685	ETH-87,313	Mixed benthic foraminifera	8375	80	8,833	8,835	159	315
	694	ETH-87,314	Mixed benthic foraminifera	8340	70	8,794	8,792	151	292
	731	ETH-102,786	Mixed benthic foraminifera	8290	60	8,730	8,729	146	268
	734	KIA-54,313	Mollusc shell	8555	35	9,098	9,091	118	251
	752	ETH-102,787	Mollusc shell	8505	70	9,033	9,022	157	309
	771	ETH-107,324	Mollusc shells	8395	70	8,855	8,861	152	303
	784	ETH-107,325	Mixed benthic foraminifera	8420	70	8,883	8,895	157	306
	800	ETH-87,315	Mixed benthic foraminifera	9200	70	9,879	9,877	170	298
MSM46-24-01-GC	39	KIA-54,314	Mollusc shell	1068	23	566	564	70	151
	119	ETH-102,156	Mixed benthic foraminifera	2140	50	1,632	1,634	116	234
	219	ETH-102,157	Mixed benthic foraminifera	2770	60	2,432	2,425	137	264
	306	KIA-54,315	Mollusc shell	2950	27	2,633	2,631	107	220
	456	KIA-54,316	Mollusc shell	3656	27	3,481	3,488	106	220
	469	ETH-102,158	Mixed benthic foraminifera	4070	60	4,013	4,020	139	285
	519	ETH-102,159	Mixed benthic foraminifera	4290	60	4,297	4,310	139	284
	537	KIA-54,317	Mollusc shell	4358	29	4,398	4,402	121	251
	569	ETH-102,160	Mixed benthic foraminifera	4770	60	4,932	4,933	137	287
	600	KIA-54,318	Mollusc shell	4805	35	4,959	4,974	123	245
	631	ETH-102,161	Mixed benthic foraminifera	5900	70	6,187	6,195	125	246
	694	KIA-54,319	Mollusc shell	6720	35	7,107	7,101	109	212
	722.5	UOC-2521	Mollusc shells	6920	35	7,319	7,307	93	190
	729.5	UOC-2522	Mollusc shells	7000	30	7,379	7,379	90	179
	769	ETH-102,162	Mixed benthic foraminifera	7560	60	7,904	7,909	110	226
	769.5	ETH-111,073	Mollusc shell	7540	60	7,888	7,888	109	225
	814	ETH-111,074	G. auriculata	7860	70	8,229	8,222	119	232
	829	ETH-102,163	Ostracods	8620	70	9,156	9,167	137	267
	829	ETH-102,164	Mixed benthic foraminifera	8650	70	9,202	9,201	139	253
	847	ETH-111,076	Mixed benthic foraminifera	8905	90	9,493	9,496	150	327

## Supplementary Information

Below is the link to the electronic supplementary material.

Supplementary Material 1

## Data Availability

All original data is available from the supplementary file.

## References

[1] Rignot, E., Velicogna, I., van den Broeke, M. R., Monaghan, A. & Lenaerts, J. T. M. Acceleration of the contribution of the Greenland and Antarctic ice sheets to sea level rise. *Geophys. Res. Lett.* **38**, L05503. 10.1029/2011gl046583 (2011).

[2] Shepherd, A. et al. Mass balance of the Greenland ice sheet from 1992 to 2018. *Nature* **579**, 233–239. 10.1038/s41586-019-1855-2 (2020). 31822019 10.1038/s41586-019-1855-2

[3] Rasmussen, S. O. et al. A new Greenland ice core chronology for the last glacial termination. *J. Geophys. Res.* **111**10.1029/2005JD006079 (2006).

[4] Thomas, E. R. et al. The 8.2 ka event from Greenland ice cores. *Quat Sci. Rev.* **26**, 70–81. 10.1016/j.quascirev.2006.07.017 (2007).

[5] Keigwin, L. D., Jones, G. A., Lehman, S. J. & Boyle, E. A. Deglacial meltwater discharge, North Atlantic deep Circulation, and abrupt climate change. *J. Geophys. Res.* **96**, 16811–16826. 10.1029/91JC01624 (1991).

[6] Alley, R. B. et al. Holocene Climatic instability: A prominent, widespread event 8200 year ago. *Geology* **25**, 483–486. 10.1130/0091-7613(1997)025%3C0483:HCIAPW%3E2.3.CO;2 (1997).

[7] Clark, P. U. et al. Freshwater forcing of abrupt climate change during the last glaciation. *Science* **293**, 283–287. 10.1126/science.1062517 (2001). 11452120 10.1126/science.1062517

[8] Meissner, K. J. & Clark, P. U. Impact of floods versus routing events on the thermohaline circulation. *Geophys. Res. Lett.* **33**10.1029/2006GL026705 (2006).

[9] LeGrande, A. N. & Schmidt, G. A. Ensemble, water isotope–enabled, coupled general circulation modeling insights into the 8.2 ka event. *Paleoceanography* **23**10.1029/2008PA001610 (2008).

[10] Clarke, G. K. C., Bush, A. B. G. & Bush, J. W. M. Freshwater Discharge, sediment Transport, and modeled climate impacts of the final drainage of glacial lake Agassiz. *J. Clim.* **22**, 2161–2180. 10.1175/2008jcli2439.1 (2009).

[11] Morrill, C., LeGrande, A. N., Renssen, H., Bakker, P. & Otto-Bliesner, B. L. Model sensitivity to North Atlantic freshwater forcing at 8.2 ka. *Clim. Past*. **9**, 955–968. 10.5194/cp-9-955-2013 (2013).

[12] Klitgaard-Kristensen, D., Sejrup, H. P., Haflidason, H., Johnsen, S. & Spurk, M. A regional 8200 cal. Yr BP cooling event in Northwest Europe, induced by final stages of the Laurentide ice-sheet deglaciation? *J. Quat Sci.* **13**, 165–169 (1998).

[13] Barber, D. C. et al. Forcing of the cold event of 8,200 years ago by catastrophic drainage of Laurentide lakes. *Nature* **400**, 344–348. (1999).

[14] Lajeunesse, P. & St-Onge, G. The subglacial origin of the lake Agassiz–Ojibway final outburst flood. *Nat. Geosci.* **1**, 184–188. 10.1038/ngeo130 (2008).

[15] Dyke, A. S. An outline of North American deglaciation with emphasis on central and northern Canada. In Quaternary Glaciations–Extent and Chronology—Part II: North America, Developments in Quaternary Science (eds Ehlers, J. et al.) 373–424. 10.1016/S1571-0866(04)80209-4 (Elsevier, 2004).

[16] Gauthier, M. S., Kelley, S. E. & Hodder, T. J. Lake Agassiz drainage bracketed holocene Hudson Bay ice saddle collapse. *Earth Planet. Sci. Lett.* **544**10.1016/j.epsl.2020.116372 (2020).

[17] Carlson, A. E. et al. Rapid early holocene deglaciation of the Laurentide ice sheet. *Nat. Geosci.* **1**, 620–624. 10.1038/ngeo285 (2008).

[18] Carlson, A. E., Clark, P. U., Haley, B. A. & Klinkhammer, G. P. Routing of western Canadian Plains runoff during the 8.2 ka cold event. *Geophys. Res. Lett.* **36**10.1029/2009GL038778 (2009).

[19] Gregoire, L. J., Payne, A. J. & Valdes, P. J. Deglacial rapid sea level rises caused by ice-sheet saddle collapses. *Nature* **487**, 219–222. 10.1038/nature11257 (2012). 22785319 10.1038/nature11257

[20] Matero, I. S. O., Gregoire, L. J., Ivanovic, R. F., Tindall, J. C. & Haywood, A. M. The 8.2 ka cooling event caused by Laurentide ice saddle collapse. *Earth Planet. Sci. Lett.* **473**, 205–214. 10.1016/j.epsl.2017.06.011 (2017).

[21] Carlson, A. E. & Clark, P. U. Ice sheet sources of sea level rise and freshwater discharge during the last deglaciation. *Rev. Geophys.* **50**10.1029/2011RG000371 (2012).

[22] Jennings, A. E., Andrews, J. T., Pearce, C., Wilson, L. J. & Ólfasdótttir, S. Detrital carbonate peaks on the Labrador shelf, a 13–7 ka template for freshwater forcing from the Hudson Strait outlet of the Laurentide ice sheet into the subpolar Gyre. *Quat Sci. Rev.* **107**, 62–80. 10.1016/j.quascirev.2014.10.022 (2015).

[23] Lochte, A. A. et al. Labrador Sea freshening at 8.5 ka BP caused by Hudson Bay ice saddle collapse. *Nat. Commun.* **10**10.1038/s41467-019-08408-6 (2019). 10.1038/s41467-019-08408-6 PMC6362222 30718573

[24] Brouard, E., Roy, M., Godbout, P. M. & Veillette, J. J. A framework for the timing of the final meltwater outbursts from glacial lake Agassiz-Ojibway. *Quat Sci. Rev.* **274**10.1016/j.quascirev.2021.107269 (2021).

[25] St-Onge, G. & Lajeunesse, P. Flood-Induced turbidites from Northern Hudson Bay and Western Hudson strait: A Two-Pulse record of lake Agassiz final outburst flood? in Submarine Mass Movements and their Consequences (Eds (eds Lykousis, V., Sakellariou, D. & Locat, J.) 129–137. 10.1007/978-1-4020-6512-5_14 (Springer, (2007).

[26] Haberzettl, T., St-Onge, G. & Lajeunesse, P. Multi-proxy records of environmental changes in Hudson Bay and Strait since the final outburst flood of lake Agassiz–Ojibway. *Mar. Geol.* **271**, 93–105. 10.1016/j.margeo.2010.01.014 (2010).

[27] Hillaire-Marcel, C., de Vernal, A. & Piper, D. J. W. Lake Agassiz final drainage event in the Northwest North Atlantic. *Geophys. Res. Lett.* **34**10.1029/2007gl030396 (2007).

[28] Lutz, B., Wiles, G. C., Lowell, T. V. & Michaels, J. The 8.2-ka abrupt climate change event in brown’s Lake, Northeast Ohio. *Quat Res.* **67**, 292–296. 10.1016/j.yqres.2006.08.007 (2007).

[29] Dyke, A. S., Moore, A. & Robertson, L. Deglaciation of North America, scale 1:7000000. Geological survey of Canada. *Open. File*. 10.4095/2143999 (2003).

[30] Shilts, W. W. Flow patterns in the central North American ice sheet. *Nature* **286** (5770), 213–218. 10.1038/286213a0 (1980).

[31] Henderson, P. J. *Provenance and Depositional Facies of Surficial Sediments in Hudson Bay, a Glaciated Epeiric Sea* (PhD, University of Ottawa, 1990).

[32] GEBCO Bathymetric Compilation Group GEBCO 2025 grid. *Br. Oceanogr. Data Centre Natl. Oceanogr. Centre NERC UK*. 10.5285/37c52e96-24ea-67ce-e063-7086abc05f29 (2025).

[33] Kerwin, M. W. A regional stratigraphic isochron (ca. 800014 C Yr B.P.) from final deglaciation of Hudson Strait. *Quat Res.* **46**, 89–98. 10.1006/qres.1996.0049 (1996).

[34] Duboc, Q., Lajeunesse, P., St-Onge, G., Moros, M. & Perner, K. Holocene sedimentary sequences from Nachvak and Saglek Fjords (Northern Labrador) as a record of deglaciation of the Torngat mountains and Hudson Bay. *Quat Sci. Rev.* **307**10.1016/j.quascirev.2023.108046 (2023).

[35] Laymon, C. A. Glacial geology of Western Hudson Strait, Canada, with reference to Laurentide ice sheet dynamics. *GSA Bull.* **104**, 1169–1177. 10.1130/0016-7606(1992)104%3C1169:GGOWHS%3E2.3.CO;2 (1992).

[36] Aylsworth, J. M. & Shilts, W. W. Surficial geology of coats and Mansel islands, Northwest territories. *Geol Surv. Can. Paper 89 – 23*. 1–26. 10.4095/131927 (1991).

[37] Ross, M., Lajeunesse, P. & Kosar, K. G. A. The subglacial record of Northern Hudson bay: insights into the Hudson Strait ice stream catchment. *Boreas* **40**, 73–91. 10.1111/j.1502-3885.2010.00176.x (2011).

[38] Josenhans, H. W. & Zevenhuizen, J. Dynamics of the Laurentide ice sheet in Hudson Bay, Canada. *Mar. Geol.* **92**, 1–26. 10.1016/0025-3227(90)90024-E (1990).

[39] Dowdeswell, J. A. & Cofaigh, Ó. Glacier-Influenced sedimentation on High-Latitude continental margins. *Geol Soc. London Special Publ.*. **203**, 1–378. 10.1144/GSL.SP.2002.203 (2002).

[40] Clarke, G. K. C., Leverington, D. W., Teller, J. T. & Dyke, A. S. Paleohydraulics of the last outburst flood from glacial lake Agassiz and the 8200BP cold event. *Quat Sci. Rev.* **23**, 389–407. 10.1016/j.quascirev.2003.06.004 (2004).

[41] Breckenridge, A., Lowell, T. V., Stroup, J. S. & Evans, G. A review and analysis of varve thickness records from glacial lake Ojibway (Ontario and Quebec, Canada). *Quat Int.* **260**, 43–54. 10.1016/j.quaint.2011.09.031 (2012).

[42] Godbout, P. M., Roy, M. & Veillette, J. J. High-resolution varve sequences record one major late-glacial ice readvance and two drainage events in the Eastern lake Agassiz-Ojibway basin. *Quat Sci. Rev.* **223**10.1016/j.quascirev.2019.105942 (2019).

[43] Godbout, P. M., Roy, M. & Veillette, J. J. A detailed lake-level reconstruction shows evidence for two abrupt lake drawdowns in the late-stage history of the Eastern lake Agassiz-Ojibway basin. *Quat Sci. Rev.* 238. 10.1016/j.quascirev.2020.106327 (2020).

[44] Roy, M., Brouard, E., Godbout, P. M. & Turcot, D. The evolution of glacial lake Barlow and its connection to lake Ojibway during the deglaciation of the south-central Laurentide ice sheet. *Quat Sci. Rev.* 372. 10.1016/j.quascirev.2025.109713 (2026).

[45] Roy, K. & Peltier, W. R. Glacial isostatic adjustment, relative sea level history and mantle viscosity: reconciling relative sea level model predictions for the U. S. East Coast with geological constraints. *Geophys. J. Int.* **201**, 1156–1181. 10.1093/gji/ggv066 (2015).

[46] Godbout, P. M., Brouard, E. & Roy, M. 1-km resolution rebound surfaces and paleotopography of glaciated North America since the last glacial maximum. *Sci. Data*. **10**10.1038/s41597-023-02566-5 (2023). 10.1038/s41597-023-02566-5 PMC10593785 37872190

[47] Smith, J. A. et al. Sub-ice-shelf sediments record history of twentieth-century retreat of pine Island glacier. *Nature* **541**, 77–80. 10.1038/nature20136 (2017). 27880756 10.1038/nature20136

[48] Chudley, T. R., Howat, I. M., King, M. D. & Negrete, A. Atlantic water intrusion triggers rapid retreat and regime change at previously stable Greenland glacier. *Nat. Commun.* **14**10.1038/s41467-023-37764-7 (2023). 10.1038/s41467-023-37764-7 PMC10115864 37076489

[49] Reed, B., Green, J. A. M., Jenkins, A. & Gudmundsson, G. H. Recent irreversible retreat phase of pine Island glacier. *Nat. Clim. Change*. **14**, 75–81. 10.1038/s41558-023-01887-y (2024).

[50] Sanford, B. V. & Grant, A. C. Paleozoic and mesozoic geology of the Hudson and Southeast Arctic platforms. *Geol Surv. Canada Open. File Rep.* **3595**10.4095/210108 (1998).

[51] Gao, C. Late history of glacial lake Agassiz in Northwestern Ontario, canada: A case study in the sandy lake basin. *Can. J. Earth Sci.* **61**10.1139/cjes-2023-0014 (2023).

[52] Roy, M. Insights on the events surrounding the final drainage of lake Ojibway based on James Bay stratigraphic sequences. *Quat Sci. Rev.* **30**, 682–692. 10.1016/j.quascirev.2010.12.008 (2011).

[53] Gao, C. & Turton, C. L. Early holocene marine incursion and a freshened Tyrrell sea in Hudson Bay Lowlands, Canada. *Quat. Sci. Rev.* **349**10.1016/j.quascirev.2024.109134 (2025).

[54] Hughes, O. L. Surficial Geology of Part of the Cochrane District, Ontario, Canada. in *International Studies on the Quaternary: Papers Prepared on the Occasion of the VII Congress of the International Association for Quaternary Research Boulder, Colorado, 1965* (Eds Wright Jr, H. E. and Frey, D. G.) 535–565. 10.1130/SPE84-p535 (Geological Society of America, 1965).

[55] Brooks, G. R. Insights into the Connaught sequence of the Timiskaming varve series from Frederick house Lake, Northeastern Ontario. *Can. J. Earth Sci.* **58**, 1268–1282. 10.1139/cjes-2020-0217 (2021).

[56] Ellison, C. R., Chapman, M. R. & Hall, I. R. Surface and deep ocean interactions during the cold climate event 8200 years ago. *Science* **312**, 1929–1932. 10.1126/science.1127213 (2006). 16809535 10.1126/science.1127213

[57] Dalton, A. S. et al. Deglaciation of the North American ice sheet complex in calendar years based on a comprehensive database of chronological data: NADI-1. *Quat. Sci. Rev.* **321**10.1016/j.quascirev.2023.108345 (2023).

[58] Shepherd, A., Wingham, D. & Rignot, E. Warm ocean is eroding West Antarctic ice sheet. *Geophys. Res. Lett.* **31**10.1029/2004GL021106 (2004).

[59] Joughin, I., Smith, B. E. & Holland, D. M. Sensitivity of 21st century sea level to ocean-induced thinning of pine Island Glacier, Antarctica. *Geophys. Res. Lett.* **37**10.1029/2010GL044819 (2010).

[60] Pritchard, H. D. et al. Antarctic ice-sheet loss driven by basal melting of ice shelves. *Nature* **484**, 502–505. 10.1038/nature10968 (2012). 22538614 10.1038/nature10968

[61] Rignot, E., Jacobs, S., Mouginot, J. & Scheuchl, B. Ice-shelf melting around Antarctica. *Science* **341**, 266–270. 10.1029/2011GL046583 (2013). 23765278 10.1126/science.1235798

[62] Paolo, F. S., Fricker, H. A. & Padman, L. Volume loss from Antarctic ice shelves is accelerating. *Science* **348**, 327–331. 10.1126/science.aaa0940 (2015). 25814064 10.1126/science.aaa0940

[63] Payne, A. J., Vieli, A., Shepherd, A. P., Wingham, D. J. & Rignot, E. Recent dramatic thinning of largest West Antarctic ice stream triggered by oceans. *Geophys. Res. Lett.* **31**10.1029/2004gl021284 (2004).

[64] Gao, F. et al. Investigating the impact of sub-ice shelf melt on Antarctica Ice Sheet spin-up and projections. *EGUsphere*,, 1–23, 1–23 (2025). 10.5194/egusphere-2025-3264 (2025).

[65] Schoof, C. Ice sheet grounding line dynamics: steady states, stability, and hysteresis. *J. Geophys. Res.* **112**10.1029/2006JF000664 (2007).

[66] Durand, G., Gagliardini, O., de Fleurian, B., Zwinger, T. & Le Meur, E. Marine ice sheet dynamics: hysteresis and neutral equilibrium. *J. Geophys. Res.* **114**, F03009. 10.1029/2008JF001170 (2009).

[67] Jamieson, S. S. R. et al. Ice-stream stability on a reverse bed slope. *Nat. Geosci.* **5**, 799–802. 10.1038/ngeo1600 (2012).

[68] Gandy, N. et al. Marine ice sheet instability and ice shelf buttressing of the minch ice Stream, Northwest Scotland. *Cryosphere* **12**, 3635–3651. 10.5194/tc-12-3635-2018 (2018).

[69] Ullman, D. J. et al. Final Laurentide ice-sheet deglaciation and holocene climate-sea level change. *Quat. Sci. Rev.* **152**, 49–59. 10.1016/j.quascirev.2016.09.014 (2016).

[70] Hijma, M. P. & Cohen, K. M. Timing and magnitude of the sea-level jump preluding the 8200 year event. *Geology* **38**, 275–278. 10.1130/G30439.1 (2010).

[71] Hijma, M. P. & Cohen, K. M. Holocene sea-level database for the Rhine-Meuse Delta, the netherlands: implications for the pre-8.2 ka sea-level jump. *Quat. Sci. Rev.* **214**, 68–86. 10.1016/j.quascirev.2019.05.001 (2019).

[72] Li, Y. X., Törnqvist, T. E., Nevitt, J. M. & Kohl, B. Synchronizing a sea-level jump, final lake Agassiz drainage, and abrupt cooling 8200 years ago. *Earth Planet. Sci. Lett.* **315–316**, 41–50. 10.1016/j.epsl.2011.05.034 (2012).

[73] Törnqvist, T. E. & Hijma, M. P. Links between early holocene ice-sheet decay, sea-level rise and abrupt climate change. *Nat. Geosci.* **5**, 601–606. 10.1038/ngeo1536 (2012).

[74] Lawrence, T., Long, A. J., Gehrels, W. R., Jackson, L. P. & Smith, D. E. Relative sea-level data from Southwest Scotland constrain meltwater-driven sea-level jumps prior to the 8.2 Kyr BP event. *Quat. Sci. Rev.* **151**, 292–308. 10.1016/j.quascirev.2016.06.013 (2016).

[75] Rush, G. et al. The magnitude and source of meltwater forcing of the 8.2 ka climate event constrained by relative sea-level data from eastern Scotland. *Quat. Sci. Adv.* **12**10.1016/j.qsa.2023.100119 (2023).

[76] Rohling, E. J. et al. Differences between the last two glacial maxima and implications for ice-sheet, δ18O, and sea-level reconstructions. *Quat. Sci. Rev.* **176**, 1–28. 10.1016/j.quascirev.2017.09.009 (2017).

[77] Morrill, C., Ward, E. M., Wagner, A. J., Otto-Bliesner, B. L. & Rosenbloom, N. Large sensitivity to freshwater forcing location in 8.2 ka simulations. *Paleoceanography* **29**, 930–945. 10.1002/2014pa002669 (2014).

[78] Hoffman, J. S. et al. Linking the 8.2 ka event and its freshwater forcing in the Labrador Sea. *Geophys. Res. Lett.* **39**10.1029/2012GL053047 (2012).

[79] Hardy, L. La déglaciation et les épisodes Lacustre et Marin Sur les versants de La partie québécoise des basses Terres de La Baie de James. *Geogr. Phys. Quat*. **31**, 261–273. 10.7202/1000277ar (1977).

[80] Hillaire-Marcel, C., Occhietti, S. & Vincent, J. S. Sakami moraine, quebec: A 500-km-long moraine without Climatic control. *Geology* **9**, 210–214. 10.1130/0091-7613(1981)9%3C210 (1981). :Smqakm>2.0.Co;2

[81] Couette, P. O. et al. Evidence for an extensive ice shelf in northern Baffin Bay during the Last Glacial Maximum. *Commun. Earth Environ.* 3, 10.1038/s43247-022-00559-7 (2022).

[82] Falconer, G., Andrews, J. T. & Ives, J. D. Late-Wisconsin end moraines in Northern Canada. *Science* **147**, 608–610. 10.1126/science.147.3658.608 (1965). 17783266 10.1126/science.147.3658.608

[83] Briner, J. P., Bini, A. C. & Anderson, R. S. Rapid early holocene retreat of a Laurentide outlet glacier through an Arctic Fjord. *Nat. Geosci.* **2**, 496–499. 10.1038/ngeo556 (2009).

[84] Young, N. E., Briner, J. P., Rood, D. H. & Finkel, R. C. Glacier extent during the younger Dryas and 8.2-ka event on Baffin Island, Arctic Canada. *Science* **337**, 1330–1333. 10.1126/science.1222759 (2012). 22984068 10.1126/science.1222759

[85] Young, N. E. et al. Age of the Fjord Stade moraines in the Disko Bugt region, Western Greenland, and the 9.3 and 8.2 ka cooling events. *Quat. Sci. Rev.* **60**, 76–90. 10.1016/j.quascirev.2012.09.028 (2013).

[86] Young, N. E. et al. Deglaciation of the Greenland and Laurentide ice sheets interrupted by glacier advance during abrupt coolings. *Quat. Sci. Rev.* **229**, 106091. 10.1016/j.quascirev.2019.106091 (2020).

[87] Young, N. E. et al. Pulsebeat of early holocene glaciation in Baffin Bay from high-resolution beryllium-10 moraine chronologies. *Quat. Sci. Rev.* **270**, 107179. 10.1016/j.quascirev.2021.107179 (2021).

[88] Schulz-Bull et al. *Response of (C)oastal (E)cosystems To Biogeochemical and Hydrographic Changes in Eastern (CA)nadian (S)eas during Holocene and Anthropocene. MARIA S. MERIAN-Berichte Cruise MSM* Vol. 46, 1–71 10.48433/cr_msm46(Leibniz Institute for Baltic Sea Research Warnemünde, 2022).

[89] St-Onge, G., Mulder, T., Francus, P. & Long, B. Elsevier, Chapter two continuous physical properties of cored marine sediments. in: *Developments in Marine Geology*, 63–98.10.1016/S1572-5480(07)01007-X (2007).

[90] Andrews, J. T. & Stravers, J. A. Magnetic susceptibility of late quaternary marine sediments, Frobisher Bay, N.W.T.: an indicator of changes in provenance and processes. *Quat. Sci. Rev.* **12**, 157–167. 10.1016/0277-3791(93)90050-V (1993).

[91] Stoner, J. S., Channell, J. E. & Hillaire-Marcel, C. The magnetic signature of rapidly deposited detrital layers from the deep Labrador sea: relationship to North Atlantic Heinrich layers. *Paleoceanography* **11**, 309–325. 10.1029/96PA00583 (1996).

[92] Stoner, J. S. & St-Onge, G. Magnetic Stratigraphy in Paleoceanography: Reversals, Excursions, Paleointensity, and Secular Variation. in *Developments in Marine Geology* (Eds. Hillaire–Marcel, C. and De Vernal, A.), 99–138. 10.1016/S1572-5480(07)01008-1 (Elsevier, 2007).

[93] Dypvik, H. & Harris, N. B. Geochemical facies analysis of fine-grained siliciclastics using Th/U, Zr/Rb and (Zr + Rb)/Sr ratios. *Chem. Geol.* **181**, 131–146. 10.1016/S0009-2541(01)00278-9 (2001).

[94] Croudace, I. W. & Rothwell, R. G. (eds) *Micro-XRF Studies of Sediment Cores: Applications of a non-destructive Tool for the Environmental Sciences* Vol. 9 https://link.springer.com/book/10.1007/978-94-017-9849-5 (Springer, 2015).

[95] Duboc, Q., St-Onge, G. & Lajeunesse, P. Sediment records of the influence of river damming on the dynamics of the Nelson and Churchill Rivers, Western Hudson Bay, Canada, during the last centuries. *Holocene* **27**, 712–725. 10.1177/0959683616670465 (2017).

[96] King, J. W., Banerjee, S. K. & Marvin, J. A new rock-magnetic approach to selecting sediments for geomagnetic paleointensity studies: application to paleointensity for the last 4000 years. *J. Geophys. Res.* **88**, 5911–5921. 10.1029/JB088iB07p05911 (1983).

[97] Alley, R. B. & MacAyeal, D. R. Ice-rafted debris associated with binge/purge oscillations of the Laurentide ice sheet. *Paleoceanography* **9**, 503–511. 10.1029/94PA01008 (1994).

[98] Andrews, J. T. Icebergs and iceberg rafted detritus (IRD) in the North atlantic: facts and assumptions. *Oceanography* **13**, 100–108. 10.5670/oceanog.2000.19 (2000).

[99] Stuiver, M. & Reimer, P. J. Extended 14 C data base and revised CALIB 3.0 14 C age calibration program. *Radiocarbon* **35**, 215–230. 10.1017/S0033822200013904 (1993).

[100] Heaton, T. J. et al. Marine20—The marine radiocarbon age calibration curve (0–55,000 cal BP). *Radiocarbon* **62**, 779–820. 10.1017/RDC.2020.68 (2020).

[101] Pieńkowski, A. J., Coulthard, R. D. & Furze, M. F. A. Revised marine reservoir offset (∆R) values for molluscs and marine mammals from Arctic North America. *Boreas* **52**, 145–167. 10.1111/bor.12606 (2023).

[102] Vickers, K. J., Ward, B. C., Utting, D. J. & Telka, A. M. Deglacial reservoir age and implications, Foxe Peninsula, Baffin Island. *J. Quat Sci.* **25**, 1338–1346. 10.1002/jqs.1419 (2010).

[103] Lewis, C. F. M., Miller, A. A. L., Levac, E., Piper, D. J. W. & Sonnichsen, G. V. Lake Agassiz outburst age and routing by Labrador current and the 8.2 cal Ka cold event. *Quat. Int.* **260**, 83–97. 10.1016/j.quaint.2011.08.023 (2012).

[104] Ross, M., Utting, D. J., Lajeunesse, P. & Kosar, K. G. A. Early holocene deglaciation of Northern Hudson Bay and Foxe channel constrained by new radiocarbon ages and marine reservoir correction. *Quat. Res.* **78**, 82–94. 10.1016/j.yqres.2012.03.001 (2012).

[105] Rashid, H., Piper, D. J. W., Lazar, K. B., McDonald, K. & Saint-Ange, F. The holocene Labrador current: changing linkages to atmospheric and oceanographic forcing factors. *Paleoceanography* **32**, 498–510. 10.1002/2016PA003051 (2017).

[106] Caron, M. et al. Holocene chronostratigraphy of Northeastern Baffin Bay based on radiocarbon and palaeomagnetic data. *Boreas* **48**, 147–165. 10.1111/bor.12346 (2019).

[107] Allan, E. et al. Insolation vs. meltwater control of productivity and sea surface conditions off SW Greenland during the holocene. *Boreas* **50**, 631–651. 10.1111/bor.12514 (2021).

[108] Blaauw, M. & Christen, J. A. Flexible paleoclimate age-depth models using an autoregressive gamma process. *Bayesian Anal.* **6**, 457–474. 10.1214/11-BA618 (2011).

